# BioFlx Pediatric Crowns: Current Evidence on Clinical Outcomes and Material Properties

**DOI:** 10.3390/children12101281

**Published:** 2025-09-23

**Authors:** Sanaa N. Al-Haj Ali

**Affiliations:** Department of Orthodontics and Pediatric Dentistry, College of Dentistry, Qassim University, Qassim 52571, Saudi Arabia; dr.sanaa.alhajali@qudent.org or s.alhajali@qu.edu.sa

**Keywords:** crowns, BioFlx, pediatric dentistry, treatment outcome, tooth, deciduous, zirconia

## Abstract

BioFlx crowns represent an innovative hybrid resin polymer-based alternative for pediatric full-coverage restorations, addressing the clinical dilemma between durable-but-unaesthetic stainless steel crowns (SSCs) and technique-sensitive zirconia crowns. This narrative review synthesizes current evidence of BioFlx crowns’ mechanical properties, clinical performance, and material characteristics through a comprehensive literature search across PubMed, Scopus, and Web of Science from August through September 2025. The search identified 18 studies comprising four randomized controlled trials, two case reports/series, and twelve in vitro studies. In vitro analyses demonstrated favorable stress distribution under physiological loads (≤311 N) with notable brand-dependent performance variations. NuSmile BioFlx exhibited greater wear than zirconia, but superior wear resistance compared to SSCs, while Kids-e-Dental BioFlx crowns demonstrated less crown wear relative to zirconia, with both brands causing less antagonist wear than zirconia. BioFlx showed intermediate fracture resistance, comparable surface roughness to SSCs but higher than zirconia, and intermediate marginal gaps. Resin cements demonstrated superior retention compared to manufacturer-recommended glass ionomer and resin-modified glass ionomer cements. Clinical studies with a 12 month follow-up demonstrated 92–98% retention rates compared to 100% for SSCs, with significantly higher patient satisfaction and reduced plaque accumulation versus SSCs. However, a failure rate of 6.7% was observed. Color change values were lower than those of zirconia crowns; however, they remained clinically unacceptable (ΔE > 3.3), and stain resistance was lower than that of SSCs. Marginal integrity remained clinically acceptable, though some anatomic form deterioration occurred over time. Case reports highlighted clinical utility in nickel-allergic patients and for masking silver diamine fluoride discoloration. BioFlx crowns represent a clinically valuable esthetic alternative in pediatric dentistry, though evidence remains limited by recent market introduction, brand-specific performance variations (NuSmile vs. Kids-e-Dental), anterior tooth applicability constraints, and contraindications in bruxism and for the Hall technique. Future randomized controlled trials with ≥2 year follow-up periods are imperative to establish long-term performance. Until such evidence emerges, BioFlx crowns represent a viable clinical option for esthetically sensitive cases and nickel-allergic patients when applied with rigorous case selection.

## 1. Introduction

BioFlx crowns represent a novel category of preformed, esthetic, full-coverage pediatric restorations designed as a flexible, durable, self-adaptable, sterilizable, metal-free, and Bis-GMA-free alternative [1]. Their development addresses key limitations of existing competitors. Stainless steel crowns (SSCs), while valued for efficiency, durability, cost-effectiveness, and flexibility in allowing trimming and crimping for optimal fit [2], present significant esthetic limitations that reduce patient/parent satisfaction. Though generally biocompatible and corrosion-resistant, SSCs contain nickel—posing allergy concerns [3].

Among esthetic alternatives, prefabricated fiberglass mesh-reinforced composite resin crowns for primary molars, commercially known as Figaro Crowns (Woodbury, MN, USA), featured ‘flex fit’ technology, allowing for adaptation over the anatomic cervical convexity of molars. Similarly to SSCs, this enabled minimal tooth reduction but demonstrated significantly more occlusal wear, strain, and failures primarily due to crown structure fracture [4,5]. Furthermore, discouraging marginal and internal adaptation of Figaro crowns was reported [6].

Conversely, preformed zirconia crowns offer superior esthetics, biocompatibility, color stability, and mechanical properties (high compressive/flexural strength, hardness, scratch resistance) [7,8,9]. Despite these advantages, zirconia crowns face substantial limitations in pediatric dentistry. Cost barriers restrict accessibility, while ceramic rigidity complicates clinical adaptation: unlike malleable SSCs, zirconia cannot be trimmed chairside for marginal adaptation [8]. Adjustment procedures carry notable risks—occlusal/proximal modifications may compromise glaze and structural integrity, while improperly finished surfaces accelerate antagonist wear, prohibiting their use in patients with bruxism [8,9,10]. Preparation requirements necessitate aggressive tooth reduction (>1 mm) [9] with inherent pulpal exposure risks in primary teeth. Cementation challenges are compounded by zirconia’s resistance to conventional etching and microcrack formation during sandblasting [8,11], extending chair time through technique-sensitive protocols. These limitations can complicate zirconia crown placement in children with behavioral management challenges [12].

To address these limitations, BioFlx crowns utilize polymer technology. Polymers are high-molecular-mass macromolecules consisting of repeating structural units derived from monomers [13]. These materials exhibit advantageous mechanical properties for dental applications: low modulus of elasticity combined with high tensile strength creates biomechanical compatibility with dentin, enabling effective load distribution while reducing stress concentrations that could lead to tooth or core structure failure [14]. BioFlx crowns leverage these polymer advantages through high-impact hybrid resin composition. This hybrid architecture enables their distinctive self-adaptation mechanism, forming temporary “dimples” at high occlusal contact points rather than conventional irreversible wear patterns, which potentially preserves crown longevity while accommodating masticatory forces. Additionally, BioFlx crowns feature enamel-comparable radiopacity (~1 mm Al equivalent) for radiographic margin evaluation, laser-marked inner surfaces for identification, and sandblasted intaglio surfaces to enhance cement retention [1].

Manufactured by NuSmile Corporation (Houston, TX, USA) [15,16] and Kids-e-Dental (Mumbai, MH, India) [1], BioFlx crowns entered clinical practice in India (2021) followed by the US market (2022).

Both brands feature monochromatic shading and sizing systems analogous to SSCs, but their indications differ: Kids-e-Dental offers comprehensive anterior and posterior coverage [1], while NuSmile provides posterior-specific designs—including dimensionally reduced first molar options (0.75 mm smaller than conventional) [15,16]. Additionally, mirroring SSCs versatility, NuSmile notes that contralateral upper crowns may substitute for lower primary molars in cases of proximal caries-related space loss, particularly with trapezoid or heart-shaped anatomy [15].

Critically, both systems employ preparation protocols that are intermediate between SSCs and zirconia crowns, requiring less tooth reduction than zirconia while maintaining SSCs-like efficiency [12]. However, NuSmile crowns require slightly more occlusal reduction than their Kids-e-Dental counterparts, along with minor buccal/lingual surface preparation [15]. This streamlined approach reduces chair time compared to zirconia placement and significantly lowers behavioral management demands [12].

BioFlx crowns share conventional indications for primary tooth full-coverage restorations: multisurface lesions, developmental defects, post-pulpectomy/pulpotomy teeth, abutments for space maintainers, and high caries-risk patients [17]. According to their manufacturers, they are particularly advantageous when prioritizing esthetics for large posterior restorations extending beyond anatomic line angles [15]. Optimal use cases include primary molars in occlusion supported by natural teeth/traditional crowns, children with Frankl behavior ratings of three to four (especially during clinicians’ initial learning curves), and time-constrained situations where zirconia placement is challenging [15].

This review synthesizes current evidence on BioFlx crowns for primary teeth, evaluating their properties and preliminary clinical performance. Given the scarcity of literature since their 2021 introduction, this narrative assessment addresses a critical knowledge gap in pediatric restorative dentistry.

## 2. Materials and Methods

This review employed a narrative synthesis approach rather than a systematic review methodology. No formal risk-of-bias assessment or quality evaluation of included studies was conducted using standardized tools (e.g., Cochrane Risk of Bias tool, ROBINS-I).

**Figure 1:**
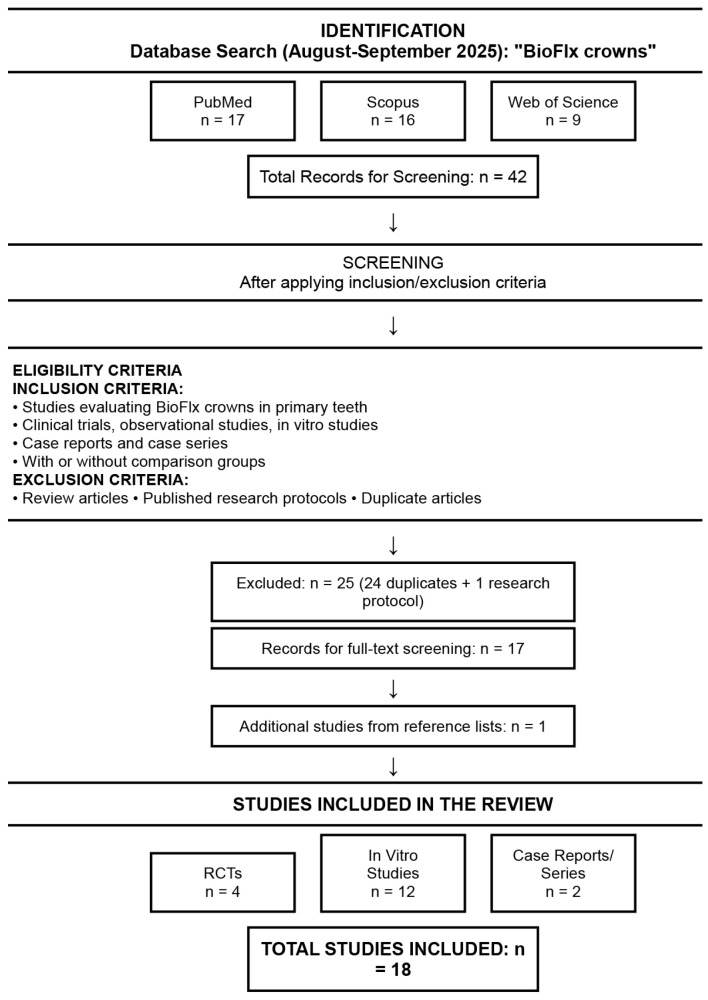
Flow diagram of literature search strategy, study selection process, and results (n = 18 studies included).

A comprehensive literature search was conducted using three electronic databases: PubMed, Web of Science, and Scopus, using the search term “BioFlx crowns” over a period of one month (August–September 2025). No restrictions were applied regarding publication date or language. Inclusion criteria were studies evaluating BioFlx crowns in primary teeth, including clinical trials, observational studies, case reports, case series, and in vitro studies. Studies could include comparisons with other crown types or restorations, or no comparison group. Exclusion criteria included review articles, published research protocols, and duplicate articles. The study selection process is summarized in Figure 1.

The search yielded 17 studies on PubMed, 16 on Scopus, and 9 on Web of Science (42 total). After removing duplicates (n = 24), and research protocols (n = 1), 17 unique studies remained for full-text screening. Reference lists of included studies were manually searched to identify additional relevant studies that were not captured in the database searches, yielding one additional study [18].

A total of 18 studies met the inclusion criteria and were included in this review, comprising 4 randomized controlled trials [19,20,21,22], two case reports or case series [3,10] and 12 in vitro studies [7,11,12,14,18,23,24,25,26,27,28,29]. As detailed in Figure 1, these studies were analyzed to synthesize current evidence on BioFlx crown properties, clinical performance, and comparative outcomes.

## 3. Results

### 3.1. In Vitro Studies

Twelve in vitro studies were analyzed: of these, six studies focused on mechanical properties (shear bond strength) and stress analysis using finite element analysis (Table 1). Three studies assessed wear resistance and surface characteristics (roughness or color stability) (Table 2). Fracture resistance and retention were assessed in two studies each (Table 3).

### 3.2. Stress Distribution and Deformation

Finite element analysis revealed variations in stress distribution across loading conditions (Table 1). Under 245 N axial loading, Lath et al. [14] reported that BioFlx crowns exhibited the lowest maximum stress when the core tooth material was glass ionomer compared to zirconia and SSCs. Kumari et al. [25] similarly demonstrated that BioFlx crowns demonstrated the least stress distribution compared to zirconia and SSCs at 45° loading. Deolikar et al. [18] further supported the trend of lower stress in BioFlx, reporting significantly lower von Mises stress in BioFlx crowns and underlying dentin versus zirconia across perpendicular and lateral loads. Conversely, Ninawe et al. [26] documented higher stress in BioFlx versus zirconia under 330 N vertical loading. In terms of deformation, Lath et al. [14] noted near-identical deformation across materials at 90° loading, with directional loading mostly influencing deformation rather than material-specific responses. Kumari et al. [25] confirmed these findings at 45° loading. However, Deolikar et al. [18] observed greater crown deformation in BioFlx compared to zirconia under all tested loading conditions (0°, 45°, 90°).

### 3.3. Cement Bond Strength

Shear bond strength testing by Singh et al. [26] showed comparable performance between BioFlx and SSCs with glass ionomer cement (GIC), and comparable performance between BioFlx crowns with GIC and resin modified glass ionomer cement (RMGI); however, with RMGI, SSCs exhibited significantly higher bond strength than BioFlx. Kumari et al. [25] demonstrated that BioFlx crowns with GIC demonstrated the highest cement stress compared to zirconia and SSCs (Table 1).

### 3.4. Wear Resistance and Surface Properties

Three studies investigated wear resistance and surface characteristics: roughness and/or color stability of BioFlx crowns compared to zirconia and SSCs under simulated aging conditions (Table 2). Of those, two studies compared BioFlx with zirconia crowns and one compared them with SSCs.

Kale et al. [28] (testing Kids-e-dental BioFlx) reported lower crown wear than zirconia post-aging, while Abdou et al. [11] (testing NuSmile BioFlx) observed higher BioFlx wear. Both studies agreed BioFlx caused less antagonist wear. Metwally et al. [29] noted that NuSmile BioFlx crowns outperformed SSCs in wear resistance after 100,000 chewing cycles.

In terms of color stability and surface roughness, BioFlx crowns demonstrated variable performance. Kale et al. [28] documented significantly less color change in BioFlx versus zirconia post-aging. Conversely, Abdou et al. [11] observed no significant color differences between Bioflx and zirconia materials after aging and beverage exposure. Surface roughness was significantly higher in BioFlx than zirconia, while Metwally et al. [29] reported comparable roughness between BioFlx and SSCs.

### 3.5. Fracture Resistance, Marginal Gap, and Retention

Abo-Elsoud et al. [7] found that SSCs demonstrated the highest fracture resistance, exceeding both BioFlx and zirconia crowns. BioFlx exhibited intermediate fracture resistance. On the contrary, Kale et al. [28] demonstrated that BioFlx crowns maintained their strength after aging simulations, while zirconia showed significant degradation. Marginal gap analysis by Abo-Elsoud et al. [7] identified zirconia as having the largest gaps, followed by BioFlx and SSCs. Retention studies indicated that SSCs generally provided the highest retention forces [19,20], though Morsy et al. [24] reported that BioFlx surpassed zirconia significantly when cemented with RMGI. Notably, BioFlx outperformed both SSCs and Zirconia when self-adhesive resin cement was used (Table 3) [23].

### 3.6. Clinical Studies

Six clinical investigations (four randomized controlled clinical trials (RCTs), one case series, one case report) evaluated BioFlx crowns over 3–12 months (Table 4). Three RCTs compared BioFlx crowns to SSCs [19,20,22], and one compared them to SSCs and zirconia crowns [21]. Notably, all clinical studies and case reports used GIC for BioFlx crowns except Singh et al. [19] who compared BioFlx with resin cement versus SSCs with GIC.

Results from randomized trials demonstrated high retention rates for BioFlx crowns (92–98%), though SSCs achieved superior retention (100%) at 12 month follow-ups [19,20]. Notably, Sunder et al. [22] reported two failed cases with BioFlx crowns, while no failed cases were reported for SSCs, marking significant differences in terms of success between both crown types. Marginal integrity remained clinically acceptable for both crown types (SSCs: 96%, BioFlx: 92%), with no significant differences [19,20]. BioFlx crowns outperformed SSCs in patient satisfaction [19,22], and effectively masked silver diamine fluoride discoloration in one report [2].

Plaque accumulation was lowest with zirconia crowns, followed by BioFlx, then SSCs, though differences were not statistically significant [21]. BioFlx crowns exhibited greater anatomic form deterioration/surface integrity deterioration at 6–12 months compared to SSCs [20,22] and higher crown substance loss at 6 months [21]. However, both crown types maintained ideal occlusion, caused no opposing tooth wear, showed no secondary caries, and demonstrated clinically acceptable marginal discoloration [20]. SSCs demonstrated greater resistance to pre-cementation dislodgement (88% vs. 80% snap fit) and approximately 1 min shorter placement time in one study [20], and 2 min longer than BioFlx crowns in another [22], with the difference in the latter study being significant. Postoperative pain was comparable across all crown types [21]. One RCT noted higher crown substance loss in BioFlx crowns at 6 months [21], while another RCT reported significantly better staining resistance of SSCs compared to BioFlx crowns at 12 months [22].

Case reports documented successful esthetic outcomes, including color stability and effective masking of silver diamine fluoride discoloration and suitability for situations like nickel-allergic patients rendering SSCs or metallic restorations contraindicated [3,10].

## 4. Discussion

The emerging literature positions BioFlx crowns as a promising alternative in pediatric restorative dentistry, effectively bridging the gap between traditional SSCs and zirconia crowns. However, it is important to note that BioFlx crowns are not yet an ideal preformed esthetic solution, particularly due to their limited availability for anterior teeth—where esthetic considerations are paramount. Furthermore, the manufacturers emphasize important clinical limitations, including that they cannot be crimped and allow for only limited contouring due to elastic memory properties, restricting chairside adaptation [1]. Without try-in options, meticulous cleaning is required to remove contaminants before cementation [10]. Furthermore, these crowns are contraindicated for the Hall technique and in cases of bruxism [1], which are clinical scenarios where SSCs maintain superiority. When addressing space loss, greater circumferential tooth reduction may be required to accommodate appropriately sized crowns, as their width cannot be altered with pliers like traditional SSCs [1]. This narrative review synthesizes current evidence regarding BioFlx crowns’ clinical performance and biomechanical properties to provide a comprehensive assessment of their role in contemporary pediatric dentistry. It is noteworthy that future systematic reviews of BioFlx crowns should incorporate formal quality assessment and risk-of-bias evaluations of included studies to strengthen evidence synthesis.

According to Ludovichetti et al. [2], pediatric crowns should be able to withstand masticatory forces, show biocompatibility, facilitate oral hygiene, present high bonding strength, and not cause damage to the antagonist teeth. In vitro finite element analysis studies predominantly indicate favorable stress distribution in BioFlx crowns, compared to conventional alternatives (zirconia and SSCs) under varied loading conditions (up to 330 N) and angles [14,18,25]. While Ninawe et al. [26] reported higher stress concentrations in BioFlx versus zirconia under 330 N vertical loading, the broader evidence suggests BioFlx crowns exhibit clinically sufficient mechanical resilience. This is further supported by physiological maximum bite force data: systematic reviews report maximum force averages of 246.2 N in primary dentition and 311 N in mixed dentition (range: 255–379 N) [30], with both means below the 330 N threshold, where stress discrepancies emerged. Consequently, Ninawe et al. [26]’s observation may have limited clinical applicability, irrespective of occlusal status (normal/malocclusion) [31].

Regarding deformation behavior, studies generally show near-identical deformation across materials [3,7], which is also promising; though Deolikar et al. [18] noted greater deformation in BioFlx, the absolute differences (0.0022 mm vs. 0.0046 mm) fall below thresholds for clinical significance and were not statistically analyzed in their study.

Regarding luting protocols, both BioFlx manufacturers permit either GIC or RMGI without preference—a position initially supported by Singh et al. [27], who reported comparable performance between BioFlx and SSCs with GIC, and equivalent outcomes for BioFlx using both GIC and RMGI. However, Kumari et al. [25] identified cement-dependent vulnerabilities, demonstrating significantly higher cement stress in GIC-cemented BioFlx crowns versus zirconia and SSCs under identical conditions perhaps carrying greater debonding risk. This was substantiated by retention studies showing material-specific interactions: BioFlx crowns cemented with RMGI exhibited significantly superior retention compared to zirconia [24], while self-adhesive resin cement enabled BioFlx to outperform SSCs in retention testing [23]. Notably, resin cement is not endorsed among the luting cement options for BioFlx crowns by either manufacturer (NuSmile or Kids-e-dental). Collectively, these findings indicate that while manufacturers endorse cement flexibility, optimal BioFlx performance appears contingent on cement selection. This underscores the need for longitudinal clinical studies to establish standardized cementation protocols.

Wear resistance studies reveal divergent brand-specific outcomes: Kids-e-Dental BioFlx crowns demonstrated less wear than zirconia [28], whereas NuSmile BioFlx exhibited greater wear than zirconia [11] and higher resistance than SSCs [29]. This divergence highlights potential brand-specific performance variations, mirroring differences observed among zirconia manufacturers [9]. Crucially, both BioFlx crown brands caused less antagonist wear than zirconia [11,28]. The increased surface roughness in BioFlx crowns after aging and beverage exposure stems from their hybrid resin polymer composition, where acidic beverages cause resin matrix softening and erosion. Regarding color stability, despite demonstrating either less color change [8] or no difference from zirconia post-aging and beverage exposure [11], BioFlx crowns consistently reached clinically unacceptable ΔE values (>3.3) [32] in both studies—revealing an inherent discoloration propensity that warrants further investigation.

In terms of fracture resistance and marginal adaptation, BioFlx crowns demonstrated an intermediate performance profile: superior fracture resistance to zirconia [20] yet lower than SSCs [19], with aging disproportionately reducing zirconia’s fracture resistance compared to BioFlx [28]. Marginal gap measurements were better than zirconia. This can be attributed to preparation design constraints: zirconia requires chamfer finish lines to prevent brittle fracture, while BioFlx accommodates feather-edge margins. As Contrepois et al. [33] established, four parameters govern crown adaptation—finish line design, cement space, veneering process, and cementation.

The clinical evidence for BioFlx crowns remains limited, consisting of four RCTs with follow-up periods extending to one year and a single comparative study against zirconia crowns. This paucity reflects their recent introduction to the dental market. In their clinical trial, Singh et al. [19] demonstrated excellent retention rates for BioFlx crowns when cemented with resin cement, although only stainless steel crowns achieved complete retention. In contrast, Sunder et al. [34] reported two failed BioFlx crowns while no SSC failures were recorded, suggesting durability concerns that warrant longer and larger clinical studies.

These findings corroborate the in vitro retention data reported by Al Mawash et al. [23]. A critical limitation of Singh et al. [19] ‘s study is the failure to specify which BioFlx crown brand was utilized—an important consideration, given the distinct preparation protocols between manufacturers. NuSmile’s requirement for buccal and lingual surface preparation would theoretically enhance resin cement wettability and penetration, potentially improving bond strength and retention compared to minimal preparation approaches.

While BioFlx crowns demonstrated lower retention and success rates compared to SSCs [19,20,22], this difference must be weighed against their significant esthetic and patient satisfaction advantages. The small retention difference may be clinically acceptable given the substantial improvement in patient acceptance and their ability to effectively mask silver diamine fluoride discoloration [10,19], expanding esthetic treatment options for minimally invasive caries management protocols. However, the observed anatomic form deterioration [20,22] and increased crown substance loss at six months [21] warrant consideration in treatment planning, suggesting BioFlx crowns may be more susceptible to wear, supporting manufacturer recommendations against their use in patients with parafunctional habits, though both materials maintained ideal occlusion without opposing tooth wear.

Chairside time revealed conflicting findings compared to SSCs: a one minute difference favoring SSCs in one study [20], and a two minute difference favoring BioFlx crowns in another [22]. Even if BioFlx crowns require a minute longer chair time, this is manageable in single crown procedures, especially since the difference was statistically insignificant [20]. However, this time differential may become more pronounced in cases requiring multiple (back-to-back) crown preparations necessitating additional tooth preparation for BioFlx crowns compared to SSCs. Despite these procedural considerations, the comparable marginal integrity (92% vs. 96%) [19,20], absence of secondary caries or antagonist wear [20], and lower plaque accumulation than SSCs [21] suggest BioFlx crowns can achieve clinically acceptable outcomes with proper technique. BioFlx crowns offer particular value in esthetically sensitive cases where SSCs are contraindicated [3], supporting their use when esthetic outcomes are prioritized over marginal retention differences. However, the higher staining tendency compared to SSCs warrants further long-term clinical studies regarding their color stability and optimal management of clinically acceptable discoloration. From a clinical perspective, BioFlx crowns offer several advantages for pediatric dentists. These crowns require intermediate preparation protocols between SSCs and zirconia; hence, they certainly reduce chairside requirements compared to zirconia crowns. This streamlined approach is particularly beneficial for managing anxious pediatric patients requesting esthetic options. Cost-effectiveness represents a significant practical consideration: while BioFlx crowns are more expensive than SSCs, they are substantially less costly than zirconia crowns, positioning them as a middle-ground option that balances esthetic outcomes with economic accessibility. The reduced chair time compared to zirconia placement may partially offset the higher material cost through improved productivity. Clinicians should note that precise size selection is critical due to limited contouring, which requires familiarity with manufacturer sizing guides.

The findings from this review demonstrate that the clinical evidence supporting BioFlx crown use in pediatric dentistry remains limited but is generally positive. Ongoing randomized controlled trials (RCTs) with one year follow-ups are currently comparing their performance to stainless steel crowns (SSCs) and zirconia crowns [34]. However, further long-term RCTs (extending to at least two years) are required to evaluate BioFlx crowns against SSCs and zirconia crowns across multiple domains: retention, wear, color stability (particularly compared to zirconia), plaque retention, gingival health, and parent/patient satisfaction using various luting cements. Future research priorities should include: (1) longitudinal studies extending beyond two years with larger sample sizes, which currently do not exist, to assess crown longevity in the primary dentition lifecycle, (2) comparative cost-effectiveness analyses, incorporating material costs, and chair time in multiple or back to back cases, (3) evaluation of the most optimum luting cements, as well as color stability, after sterilization/aging or beverage exposure, (4) mandatory specification of BioFlx crown brand (NuSmile versus Kids-e-Dental) in future studies, as not all studies in this review addressed this information within their full text, which will facilitate the detection of brand-specific performance differences, (5) development and testing of anterior crown designs where esthetic demands are highest, and (6) assessment of parental and child satisfaction scores using validated instruments compared to existing crown options. Until such evidence emerges, BioFlx crowns represent a valuable esthetic alternative in appropriately selected cases, with cement selection and careful consideration of contraindications (such as bruxism and the Hall technique) being crucial determinants of success.

## 5. Conclusions

BioFlx crowns represent a clinically viable esthetic alternative to stainless steel crowns and zirconia in pediatric dentistry. However, evidence remains limited by the recent market introduction and significant brand-specific performance variations between NuSmile and Kids-e-Dental crowns, particularly in wear resistance, surface characteristics, and preparation requirements. Critical limitations include anterior tooth applicability constraints, contraindications in bruxism/Hall technique, and propensity towards discoloration. Clinical performance shows retention rates of 92–98% with documented failure cases (6.7% at 12 months) versus 100% for stainless steel crowns, alongside statistically significant inferiority in staining resistance and surface integrity at 12 months. Cement selection critically influences retention, with resin cements showing favorable performance despite lacking manufacturer endorsement. Future randomized controlled trials with ≥2 year follow-up periods and larger sample sizes are essential to validate long-term performance across different brands and establish standardized clinical guidelines. Until then, BioFlx crowns are recommended for esthetically sensitive cases, especially patients with nickel allergies, requiring careful case selection and optimized cement choice.

## Figures and Tables

**Table 1:** Mechanical Properties and Stress Analysis of BioFlx Crowns.

Study	Year	Crown Types	Test Method	Key Findings	Clinical Significance
Lath et al. [14]	2024	BioFlx (Kids e dental), SSCs, Zirconia	Load bearing capacity/shear stresses through FEA, 245 N load, multiple angles	-Maximum axial load stress: BioFlx: 14.009 MPa (lowest stress); SSCs: 39.331 MPa; Zirconia: 40.91 MPa. -All three crown types showed similar deformation patterns under simulated occlusal forces. At 90° loading, deformation was nearly identical (5.97–5.98 mm). SSCs deformed more at 45° loading (6.527 mm vs. ~5.44 mm for ZCs/BioFlx), while ZCs and BioFlx deformed more at 0° loading (~6.47 mm vs. 5.452 mm for SSCs). Load direction influenced deformation more than material type.	BioFlx crowns with a GI core material, can withstand maximum loads and could be a viable option for clinical use. [14]
Deolikar et al. [18]	2024	BioFlx vs. Zirconia	FEA (von Mises stress/deformation), lateral and perpendicular forces of 245 N	Lower stress in BioFlx crowns/dentin across all load angles. Higher deformation in BioFlx vs. zirconia.	BioFlx crowns can be a suitable alternative to zirconia crowns and traditional stainless steel crowns.
Singh et al. [27]	2025	BioFlx (Kids e dental), SSCs with bands	Shear bond strength testing using GIC/RMGIC cements	SSCs + RMGI: 0.908 ± 0.20 MPa; BioFlx + GIC: 0.506 ± 0.25 MPa; SSCs + GIC: 0.405 ± 0.24 MPa; BioFlx + RMGI: 0.362 ± 0.21.	SSCs perform statistically better using RMGI than GIC; they also perform better than BioFlx with RMGI; however, Bioflx crowns’ performance is statistically comparable with both cements.
Ninawe et al. [26]	2025	BioFlx (Kids e dental), Zirconia, Graphene	Stress distribution and deformation capacity using FEA, 330 N vertical loading	Zirconia: 368.3 MPa (best); BioFlx: 520.92 MPa; Graphene: 555.69.	BioFlx shows moderate stress resistance and more deformation than zirconia crowns.
Waly et al. [12]	2025	BioFlx with different cements (GIC, RMGI, self-cure resin cement)	FEA cement comparison	Conventional GIC best performance followed by resin cement; RMGI showed highest deformation.	Cement selection affects biomechanical behavior, with one having a higher modulus of elasticity being preferable.
Kumari et al. [25]	2025	BioFlx, Zirconia, PEEK, SSCs, polycarbonate, Edelweiss	FEA, 100–330 N multiple loading	For posterior crowns under 45° oblique loading, Zirconia exhibited the highest overall stress distribution (286.90 MPa), followed by SSCs (267.80 MPa) and BioFlx (236.11 MPa). BioFlx showed the highest cement stress (214.45 MPa), while all crown types demonstrated similar deformation values (~30.17–30.21 mm).	Zirconia remains the preferred choice for long-term restorations requiring maximum strength, while BioFlx offers a viable alternative with enhanced biocompatibility and reduced stress distribution on tooth structure. The lower overall stress exhibited by BioFlx may be beneficial for compromised teeth or when minimizing stress concentration is clinically desired.

FEA: Finite element analysis, SSCs: Stainless steel crowns, RMGI: resin modified glass ionomer cement.

**Table 2:** Wear Resistance and Surface Properties (roughness/color stability) of BioFlx Crowns.

Study	Year	Crown Types	Aging Protocol	Wear Results	Surface Properties
Kale et al. [28]	2025	BioFlx (Kids-e-dental), Zirconia	5000 thermocycles + 120,000 mechanical load (chewing simulation)	BioFlx crowns subjected to aging exhibited significantly less volumetric wear of crown material (0.78 ± 0.36 mm) compared to zirconia crowns (1.27 ± 0.48 mm^3^), and significantly less wear of antagonist (0.59 ± 0.26 vs. 1.42 ± 0.49 mm^3^).	BioFlx crowns had significantly less mean color change values after aging than zirconia crowns (ΔE = 9.81 vs. 12.81); although both crown forms demonstrated clinically unacceptable discoloration.
Abdou et al. [11]	2025	BioFlx (NuSmile), Zirconia	5000 thermocycles + 75,000 times occlusal load (50 N) (chewing simulation)	BioFlx crowns demonstrated significantly higher volume loss than zirconia crowns (0.095 ± 0.091 vs. 0.039 ± 0.046) but significantly lower volume loss of antagonist (0.095 ± 0.091 vs. 0.534 ± 0.163).	No significant color difference between crown materials following aging and exposure to beverages (ΔE = 12.106 ± 6.216 zirconia vs. 13.864 ± 3.480 BioFlx). BioFlx: demonstrated significantly higher roughness compared to zirconia crowns (1.148 ± 0.832 vs. 0.185 ± 0.17).
Kumari et al. [29]	2025	BioFlx (NuSmile), SSCs	Vertical loading, 50 N up to 100,000 chewing cycles	BioFlx crowns (0.039 ± 0.046 mm^3^) experienced significantly lower wear volumes compared to SSCs (0.095 ± 0.091 mm^3^).	Surface roughness comparable between groups (SSCs: 0.487, BioFlx: 0.466 μm).

SSCs: Stainless steel crowns, ΔE: color change value.

**Table 3:** Fracture resistance, marginal gap and retention of BioFlx Crowns.

Study	Year	Crown Types	Test Conditions	Outcome
1-Fracture resistance and marginal gap studies				
Abo-Elsoud et al. [7]	2024	BioFlx (NuSmile), Zirconia, SSCs	After thermomechanical aging	Fracture resistance: Stainless steel crowns demonstrated the highest fracture resistance (3062.14 ± 408.97 μm), followed by Bioflx crowns (2403.44 ± 92.65 μm), while Zirconia crowns showed the lowest (1286.30 ± 91.56 μm). Marginal gap: Zirconia crowns (62.6 ± 2.6 μm) exhibited the largest average marginal gap, followed by Bioflx (58.8 ± 6.5 μm) and stainless steel crowns (50.0 ± 1.6 μm). Failure mode: BioFlx and SSCs: Surface deformation and microperforations; Zirconia: Fracture lines.
Kale et al. [28]	2025	BioFlx (Kids-e-dental), Zirconia	Before/after aging	BioFlx: Maintained adequate fracture strength after aging (1717.9 ± 328.3); Zirconia: experienced a highly significant decline in fracture strength after aging (1532.0 ± 276.2)
2-Retention studies				
Al Mawash et al. [23]	2025	BioFlx (NuSmile), SSCs	Resin dies, various cements (GIC, RMGI, self-adhesive resin cement, and polycarboxylate cement).	The SSCs exhibited significantly greater retention strength than BFCs across all cements, except the self-adhesive resin cement group where BioFlx crowns demonstrated superior retention (657.72 ± 20.60 N vs. 560.29 ± 8.74 N).
Morsy et al. [24]	2025	BioFlx (NuSmile), Zirconia, SSCs	Natural primary teeth, thermocycling (2000 cycles) and luted with either GIC or RMGI.	Stainless steel crowns exhibited the highest retention regardless of the cement. Bioflx crowns outperformed Zirconia crowns (Bioflx: GIC = 138.11 ± 30.87 N, RMGI = 218.11 ± 34.61 N; Zirconia: GIC = 35.50 ± 5.14 N, RMGI = 131.78 ± 11.91 N). RMGI had greater retention values than GIC in BioFlx and zirconia crowns.

FEA: Finite element analysis, SSCs: Stainless steel crowns, GIC: Glass ionomer cement; RMGI: resin modified glass ionomer cement.

**Table 4:** Clinical Studies and case reports/case series of BioFlx Crowns.

Study	Year	Study Design	Sample Size	Crown Types	Luting Cement Used	Assessed Parameters	Follow-Up Period	Key Clinical Findings
Singh et al. [19]	2025	Split-mouth prospective RCT	40 children (80 crowns; 40 per group)	BioFlx vs. SSCs	SSCs: GIC BioFlx: resin based cement.	-Retention -Marginal integrity -Gingival health -Overall success	3, 6, 9, 12 months	-Retention: SSCs 100%, BioFlx 98%; -Marginal integrity: SSCs 96%, BioFlx 92%; -Overall success: SSC 95%, BioFlx 92% (*p* > 0.05). -BioFlx significantly higher patient satisfaction (*p* < 0.01); -Comparable gingival health
Patil et al. [20]	2024	Split-mouth prospective RCT	38 children (76 crowns; 38 per group)	BioFlx (Kids-e-dental) vs. SSCs	GIC	-Retention -Crown wear -Procedure time -Resistance to dislodgement before cementation -Wear of opposing -Occlusion -Marginal integrity and discoloration -Anatomic crown form -Secondary caries at margins.	3, 6, 12 months	-Retention: SSCs achieved 100% retention while BioFlx demonstrated 92% retention (4 crowns lost). -Crown wear: No wear observed in either group through 3 months; at 12 months, BioFlx showed 12% wear versus 4% for SSCs (*p* > 0.05). -Procedure time: SSCs averaged 12:38 min compared to 13:54 min for BioFlx. -Resistance to dislodgement before cementation: SSCs demonstrated 88% snap fit versus 80% for BioFlx, (*p* > 0.05). -Wear of opposing teeth: None in either group. -Occlusion: All crowns maintained ideal or acceptable occlusion at all periods. -Marginal integrity and discoloration: all remained clinically ideal at all periods. -Anatomic crown form: SSCs demonstrated significantly more ideal anatomic form at 6 and 12 months (96%) compared to BioFlx crowns (82–86%); remaining crowns were clinically acceptable. -Secondary caries at margins: All were clinically ideal (*p* > 0.05).
Abdelhafez and Dhar [21]	2025	Parallel RCT	75 children (25 per group)	BioFlx (NuSmile) vs. SSCs vs. Zirconia	GIC	Postoperative pain, plaque index, crown retention (debonding rate and substance loss), and gingival index	6, 12 months	-Postoperative pain: No significant differences were observed. -Plaque accumulation: SSCs demonstrated highest plaque scores, followed by BioFlx, then zirconia crowns at both evaluation periods (*p* > 0.05). -Clinical retention: SSCs followed by BioFlx crowns showed superior performance (96%) compared to zirconia crowns (92%) regarding debonding resistance at both time points (*p* > 0.05). BioFlx crowns exhibited the highest incidence of crown substance loss at the 6 month evaluation. -Gingival health: Zirconia crowns showed optimal gingival index scores (*p* > 0.05).
Sunder et al. [22]	2025	Split-mouth pilot RCT	30 children (30 per group)	SSCs vs. BioFlx	GIC	Ryge criteria, procedural time, parental satisfaction	12 months (evaluated at baseline, 1 week, 1 month, 3 months, 6 months, 12 months)	SSCs showed significantly better staining resistance and surface integrity (*p* < 0.05) at 6 and 12 months. Two BioFlx crown failures (6.7%) vs. zero SSC failures at 12 months. SSCs required longer placement time (12.3 ± 1.53 min vs. 10.32 ± 1.48 min, *p* < 0.05). Parental preference favored BioFlx crowns.
Goswami et al. [10]	2024	Case series	3 children (1 case for each)	BioFlx (Kids-e-Dental) crowns	GIC	Discoloration, dislodgement, and gingival health	6 months	Good retention, esthetic, and patient and parent satisfaction at 6 months; self-adaptation observed; excellent masking of SDF discoloration.
Ruck and Gosnell [3]	2023	Case report	1 case	BioFlx (NuSmile) and zirconia crowns for high caries-risk child with potential nickel allergy	GIC	Retention, color stability, plaque retention, and parental acceptance.	3 months	BioFlx crowns demonstrated good adaptation with minor material self-adaptation noted during placement and after 3 months. Color stability was maintained on the Bioflx crowns and plaque retention was slightly higher than zirconia. Parental acceptance was equal for zirconia. Treatment was successful for addressing high caries-risk in a patient where metal restorations were contraindicated due to nickel allergy.

SSCs: Stainless steel crowns, SDF: Silver diamine fluoride, GIC: Glass ionomer cement; RMGI: resin modified glass ionomer cement; RCT: Randomized controlled trial.

## Data Availability

This article does not contain original research data. All data discussed are publicly available in the cited publications.
